# Entrepreneur Hubris, Organizational Ambidexterity, and Dynamic Capability Construction

**DOI:** 10.3389/fpsyg.2021.717245

**Published:** 2022-03-02

**Authors:** Yan Guo, Pei-Wen Huang, Chu Ciu, Shih-Chieh Fang, Fu-Sheng Tsai

**Affiliations:** ^1^Department of Accounting and Financial Management, College of Management and Economics, Tianjin University, Tianjin, China; ^2^Department of Business Management, College of Management, Cheng Shiu University, Kaohsiung, Taiwan; ^3^Department of Business Administration, National Cheng Kung University, Tainan City, Taiwan; ^4^North China University of Water Resources and Electronic Power, Zhengzhou, China; ^5^Center for Environmental Toxin and Emerging-Contaminant Research, Cheng Shiu University, Kaohsiung, Taiwan; ^6^Super Micro Mass Research and Technology Center, Cheng Shiu University, Kaohsiung, Taiwan

**Keywords:** dynamic capability, ambidexterity, managerial hubris, Wuxi Suntech, solar energy industry

## Abstract

This paper demonstrated the influences of initiation, development, turn-down, and reinitiation of the dynamic capability of an entrepreneurial firm in the solar energy industry. The focus is on the impact of entrepreneurial hubris, which may affect the decision of ambidexterity that can vitalize dynamic capability. The findings indicate that, when the major decision maker (the founder entrepreneur) has the trait of hubris, the decision-making process may be overly arbitrary, and a decision of being exploratory or exploitative alone is likely to be made. On the contrary, when the founder entrepreneur is aware of the hubris and shares decisive power, the decision of being ambidextrous as a dynamic capability is more freely achieved. This paper contributes by discovery of the cognitive-based microfoundation of entrepreneurial ventures and linkage of such microfoundation to organizational ambidexterity.

## Introduction

Teece et al. (1997) clearly point out that dynamic capability is the ability for an enterprise to establish, integrate, and restructure internal and external resources. In such a premise, dynamic capability is not only the ability to constantly update what an enterprise can do for gaining a competitive edge, but it is also a mechanism to reconfigure and integrate existing resources and capabilities to meet the ever-changing customer demands and competition (Zahra et al., 2006). Put differently, dynamic capabilities are high-level activities that help enterprises create, extend, and adjust their zero-level capabilities for enterprise survival (Winter, 2003).

Applying the aforementioned logic to the entrepreneurship management field, a start-up company begins to form a series of resources and capabilities during its growth (Newbert, 2005). When the market environment changes, however, the original resources and capabilities may not be suitable anymore with decreasing values. At such a moment, enterprises need to utilize dynamic capability to update these resources and competencies to match the turbulent environment. Thus, dynamic capability emerges and can evolve with organizational mechanisms and entrepreneurial ventures (Corner and Wu, 2012).

To research the evolution of a new venture’s dynamic capability and growth, extant studies used to examine the influences of environmental or firm-level factors (e.g., Newey and Zahra, 2009; Madsen, 2010). What is relatively lacking is the exploration of the factors at the microlevel of analysis, such as the entrepreneurs in terms of their personality, competence, and so forth. Even if there are some, those existing studies focus on the individual or microlevel factors’ positive influences (e.g., Jantunen et al., 2005; Khan and Lew, 2018). Microfoundations for a strategic capability are vital for cognition, personality, and behaviors are the bases of a firm’s capability aggregated by human capital (e.g., Petricevic, 2017). With those discussed in mind, however, there are relatively few researching the microfoundation for the dynamic capability (co-)evolution of the start-ups. Even fewer directly investigate entrepreneurs’ attribute influences on this topic. To fill this gap, we take two steps with related research purposes. We conduct a study with long-term case observation of the dynamic capability as ambidexterity in a context of a new venture’s rise and fall following Tushman and his peers regarding ambidexterity as the index of the dynamic capability in the development (Benner and Tushman, 2003; Smith and Tushman, 2005; Tushman and O’Reilly, 2007). Duncan (1976) first introduces the concept of ambidexterity into the field of management to describe the organizational capability to simultaneously exploit and explore (Duncan, 1976). Many scholars find that ambidexterity capability is similar or highly related to dynamic capability (e.g., Ren and Peng, 2021). For example, Tushman and Smith (2002) point out that the ambidexterity structure helps organizations to form exploratory and exploitative innovative capabilities, which have a major impact on organizational dynamic capabilities.

Second, we tend to identify the microfoundation for the formation of ambidexterity through the lens of characteristics of the senior management team (under the entrepreneurial condition, especially the key entrepreneurs). Because executives, especially the core entrepreneurs of the new venture, are the setters of the main strategic direction, the senior management roles, especially cognition, are the key to develop ambidexterity (Baron and Ward, 2004; Baron and Ensley, 2006; Baron, 2007, 2008). Scholars of strategic management and organizational theory believe that traits, behaviors, experiences, and values can influence the results of the strategies (Cyert and March, 1963; Hambrick and Mason, 1984; Zajac and Westphal, 1996; Finkelstein and Boyd, 1998; Finkelstein et al., 2008; Francioni et al., 2015; Ott et al., 2017). Although previous studies define that the senior executive team is key for the formation of dynamic capability, they ignore the influence that the traits of the leaders have made on the dynamic capability, especially the traits of the core entrepreneurs under the situation of the new ventures. Hubris of leaders is one such important trait of the senior executives, which represents a form of cognitive bias and can affect decision making (Tversky and Kahneman, 1982). Therefore, this study attempts to discuss how the hubris of leaders affects the development of a dynamic capability, such as ambidexterity. Mainly, we wish to explore the microfoundation of dynamic capability evolution, not following the traditional positive lens (in this study, the microfoundation is the managerial hubris as a trigger for a negative outcome of dynamic capability evolution).

## Literature Review

### Dynamic Capability

Puts forward the concept of dynamic capabilities for the first time, and Teece et al. (1997) defines the dynamic capabilities as the company’s integration, construction, and restructuring of internal and external resources and the ability to deal with the rapidly changing environment, whereas the dynamic capabilities are embedded in the enterprise’s organizational and managerial processes and manifested by the activities of the organization, culture, and leaders’ preference at the same time. Eisenhardt and Martin (2015) carry out the explanation of dynamic capability from the aspects of the organizational routines and processes and put forward that dynamic capability is the process for the enterprise to deal with or create market changes through the acquisition, integration, reconstruction, and the release of resources. Zott (2003) follows the definition of dynamic capabilities of Teece et al. (1997) and defines dynamic capabilities from the routines or processes of the enterprise’s allocated resources and considers the dynamic capabilities as a routine organizational process. Winter (2003) puts forward a hierarchical model of dynamic capability and thinks that dynamic capability is a kind of ability with a high level and is an ability for the enterprise to find new opportunities by identifying environmental changes and then to update the competitive ability or create new ability through reallocating resources and also combining with existing resources. Zahra et al. (2006) believe that dynamic capability is the ability for decision makers to restructure the company’s resources and regulations in an appropriate way, which is largely subject to the experience, motives, and skills of the key leaders of the company. In addition, they also believe that, when the external environment is unpredictable or changes very quickly, the dynamic capability is probably the most valuable, but an unstable or changing environment is not a necessary element of dynamic capabilities.

There are many theoretical and empirical studies that clearly point out that the enterprise with dynamic capabilities is faster and smarter than other competitive enterprises to make resource reconfiguration adapted to environmental changes, namely, that dynamic capability is one of the magic weapons to obtain a sustainable competitive edge to achieve good performance for the enterprise. The positive relationship between dynamic capabilities and organizational performance is also supported by the majority of scholars. For example, Zott (2003) confirms direct and indirect relationships between dynamic capabilities and performance through the use of computer simulations. He also studies the impact of dynamic capabilities on firm performance from an evolutionary perspective. He believes that dynamic capabilities have changed the enterprise’s capabilities, operating routines, and resource niches, thus affecting the enterprise’s performance. The evolution of dynamic capabilities includes three stages, namely, variation, selection, and retention, and takes part in market competition and improves the enterprise’s performance after the implementation of a specific resource configuration.

### Ambidexterity

Duncan (1976) first introduces the concept of ambidexterity into the field of management to describe organizational capabilities. This ability to simultaneously exploit and explore is called the ambidexterity capability of the organization (Duncan, 1976; Tushman and O’Reilly, 1996). The successful enterprise owns the double abilities to both effectively operate the current business and adapt to future changes if it needs to adapt to an increasingly dynamic and complex environment (Duncan, 1976; Tushman and O’Reilly, 1996; Gibson and Birkinshaw, 2004). Swift (2016) believes that exploitation and exploration based on research and development are necessary for the company to maintain a competitive edge. It is also an essential element in the company’s innovation process.

Exploitation is the activity by which an enterprise uses existing knowledge and skills to improve its products and services in the existing areas of business activities. Such activities can improve the efficiency of the use of resources, including enterprises engaging in such activities as optimization, selection, improvement, enrichment, selection, production, implementation, and so on. Exploration is the activity by which an enterprise breaks away from the existing management field and utilizes new knowledge and skills to discover new market opportunities and develop new market space. It includes activities such as search, change, adventure, experimentation, contingency, discovery, and innovation, etc. (O’Reilly and Tushman, 2008).

The rational allocation of resources and maintaining balance between exploitation and exploration are especially important for ambidexterity. Too much emphasis on exploitation and organizations will have an advantage in the short term, but they will gradually become obsolete because of the lack of new technology. Therefore, when the new market and technological innovation comes, the enterprises will eventually fall into the “ability trap” and suffer from elimination and failure due to the lack of new ability to match with the new environment. Excessive exploration leads to an “innovation trap,” putting the enterprise into a vicious circle because of great investment and uncertain return. To escape from this trap is very difficult because the process for improving investment has higher costs, and involves the dynamic process of getting worse before getting better, namely, worse-before-better (Lyneis and Sterman, 2015).

Although owning both exploitation and exploration is very difficult, so is the conversion between the two due to differences between skills that need to be exploited and techniques that need to be explored and also differences in the aspects of the strategy, organizational structure, implementation path, etc. (Swift, 2016).

Ambidexterity as the dynamic capability.

O’Reilly and Tushman (2008) propose the concept of ambidexterity in “Ambidexterity as a dynamic capability: Resolving the innovator’s Dilemma,” and they think enterprises with ambidexterity own the ability to compete in the mature market as well as to explore new products and services in the emerging market at the same time, and these abilities cannot only help the company reorganize existing assets and the ability to discover and seize new opportunities, they also are the key to driving the dynamic capability of the enterprise. They also emphasize the role of the top leadership teams of the enterprise’s organizations in the construction of the enterprise’s dynamic capabilities (O’Reilly and Tushman, 2004).

They explore how ambidexterity helps organizations to perceive and seize opportunities and restructure resources from an organizational perspective. To exploit the existing ability and explore the unknown ability of the organization as well as to make the two properly balanced are the requirements of ambidexterity, whereas to maintain the balance between exploiting and exploring activities, it needs to restructure, integrate, and construct the existing resources and capacities, which is also the requirement of dynamic capability.

According to the view of dynamic capability, enterprises breaking through the restriction of the existing paths need to be built based on the full utilization of existing resources and improvement of the utilization and exploration abilities of the existing products. The future competitive market is full of uncertainties and changes rapidly, and enterprises must have the exploratory abilities to quickly update and respond. According to the internal status and external changes of the organization, to integrate the organizational skills, resources, and strategies and then reallocate and correct them to adapt to environmental changes and obtain a competitive edge for the enterprise is the result that the dynamic capability theory has pursued and also is the premise for the construction of the organization with ambidexterity.

With the development of the ambidexterity theory, many scholars find that there are a lot of similarities between ambidexterity ability and dynamic capability. For example, Tushman and Smith (2002) point out that the ambidexterity structure is beneficial for the organization to form exploratory and exploitative innovation capabilities, which has a significant impact on organizational dynamic capabilities.

In follow-up studies, Jansen et al. (2009) clearly point out that there is a certain similarity between ambidexterity ability and dynamic capability. Ambidexterity is one of the dynamic capabilities. The proposing of the concept of ambidexterity ability is not just to distinguish and identify the two different abilities as exploration and exploitation, but more importantly, to integrate these two capabilities to achieve overall balance between them. Thus, there is no difference between ambidexterity ability and dynamic capability in the integration and allocation of resources. In addition, the organization with ambidexterity not only can exploit the existing potential, but also can explore new market opportunities, so it can be more suitable for the environment and obtain a competitive edge, which is consistent with the definition of dynamic capabilities. Therefore, more and more scholars have begun to agree with the opinion that ambidexterity ability is a kind of dynamic ability of the organization, whereas to regard ambidexterity ability as a dynamic capability further enriches ambidexterity theory and advances it to a new stage of development.

### Managerial Hubris

In the first 10 years of the 21st century, an important study has attracted the attention of scholars, and it believes that self-esteem has become common enough in the management class of large companies. The research opens new directions in the field of strategic management, introducing the concept of managerial hubris. CEO hubris is usually defined as overconfidence or pride (Hayward and Hambrick, 1997; Hiller and Hambrick, 2005).

What are the reasons causing managers to believe in this phenomenon? The related references list some factors about the source of a leader’s hubris. External factors include (1) the recent success of the company and (2) recent praise from the media. Hayward and Hambrick (1997) suggest that a company’s recent success is the main reason for manager hubris. By praising the performance and amplifying the effectiveness and control of the CEO, recent media assessment strengthens the status of the CEO and creates a celebrity effect at the same time. Internal factors include (1) CEO overbearing, (2) the sense of power of the CEO and long term in the position of authority, and (3) educational background. Bhandari and Deaves (2006) find that men with higher education are more likely to develop hubris than their peers with less education.

Managerial overconfidence is a form of cognitive bias and can affect decision making (Tversky and Kahneman, 1982). Previous research studies the impact of managerial overconfidence and its impact on the company’s decisions and their results. The contents of the research include the acquisition premium (Hayward and Hambrick, 1997), investment mistakes (Malmendier and Tate, 2005), the failure of the adventure (Hayward et al., 2006), mergers and acquisitions (Roll, 1986; Walsh, 1988; Hayward and Hambrick, 1997; Seth et al., 2002), well-known CEOs, and the entrepreneurial spirit (Hayward et al., 2006) and also includes the fact that hubris affects strategic activities and corporate performances.

Hayward et al. (2006) point out that an overconfident entrepreneur is more likely to cause his business to fail. With a mechanism of overestimation (Moore and Healy, 2008), overconfidence can lead to overly ambitious strategies that exaggerate the need for decisiveness, impulsivity, and power as well as the risk of spontaneity, the results of which can be disastrous (Picone et al., 2014). A CEO with a hubris bias often blocks the emergence of viable ideas (Shipman and Mumford, 2011) and ignores motivating their organizations to make adjustments to respond to critical feedback.

Scholars of strategic management and organization theory accept that an executive’s characteristics, behaviors, experiences, and values affect the results of strategies (Cyert and March, 1963; Hambrick and Mason, 1984; Zajac and Westphal, 1996; Finkelstein and Boyd, 1998; Finkelstein et al., 2008). Although managerial overconfidence is one of the features of senior executives, it has a potential impact on organizational capability.

Above are the relevant contents of managerial hubris, but in this case study, WST company is a new energy enterprise, and we put the study on the managers into the entrepreneurial environment to consider to study of the contents of hubris of entrepreneurs. Compared with the general enterprise development environment, the entrepreneurial environment has the characteristics of instability and rapid change. The resources, ability, organizational structure, and operational processes of the enterprise are not mature, and the enterprise is in a dynamic environment, which is more valuable and meaningful for the research. In addition, the founder is the founder of WST company, so studying in his behavior characteristics is critical.

**FIGURE 1:**
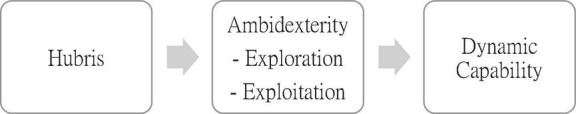
Tentative conceptual framework.

Based on the literature reviewed above, we propose a tentative conceptual framework for our further exploration of the case study. See Figure 1.

## Case Study

### Methods

In this paper, a longitudinal case study is adopted, taking the case of the solar photovoltaic enterprise WST company power as a case target. Adopting the case study method is based on the following reasons: First, data collection is mainly through cyber sources, library resources, database resources, periodicals, and other media channels to obtain literature materials, such as theories, empirical data, and successful cases that are related to the study. The form of data collection determines that this is qualitative research. Second, there are few studies on the development process of dynamic capabilities so far; thus, the case study is very appropriate. Third, this paper attempts to reveal the influence of managerial hubris on dynamic capabilities and their relationships, which has not been taken into account in previous research and is a process of mining past unknown factors. Research with a single case study can go deeper into the case investigation and analysis, more clearly show the “what,” and more clearly explain “how.” Therefore, this paper adopts the exploratory single case study method.

### Source of the Data and Its Collection

The source of the data of this study includes primary and secondary data. The collection, documentation, classification, and analysis of these data were conducted from 2000 to 2016. For the primary data, we conducted personality and telephone interviews. For the interview content, we asked them to talk about the impact of the founder’s decision making on the development of enterprises besides the business of the interviewees. The source of the secondary data is multiple historical archives of 2000–2016: the company’s brochures, internal and external announcements, public information on websites, published papers, and documents from the company. Secondary data is the source of the historical facts and also helps to sort out contextual changes. What is more, the historical texts can often help to interpret the shortcomings of the interviews.

### Case Introduction

**FIGURE 2:**
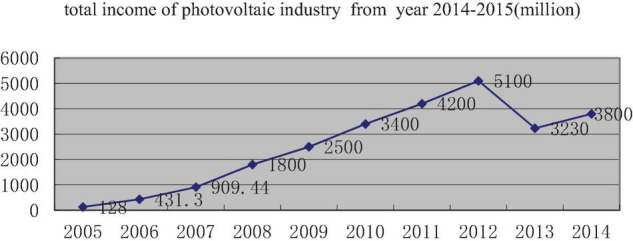
Total income of photovoltaic industry from year 2014 to 2015 (million).

**FIGURE 3:**
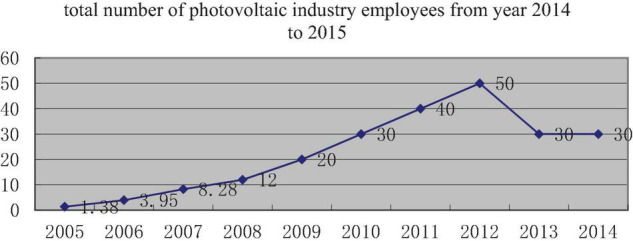
Total number of photovoltaic industry employees from year 2014 to 2015.

China’s PV industry with WST company as a representative has ridden the east wind of the world’s supportive policy of promoting PV power generation. With the development, prosperity, decline, and even following the recovery of reforming, it shows the important role of dynamic capabilities in the development of the industry. We can see the industry background from Table 1 and Figures 2, 3.

**TABLE 1:** Top 10 photovoltaic module firms around the world in 2015.

Ranking	Name of firm	Capacity/(GW)	Shipment/(GW)
1	Trina Solar	4.55	5.74
2	Artes	3.9	4.70
3	Jinko Solar	3.79	4.51
4	JA Solar	3.38	3.67
5	Presta QCells	3.2	3.3
6	First solar	2.51	2.9
7	Yingli Group Co.	2.35	2.447
8	GCL Solar	3.7	2.1
9	WST company	1.80	1.30
10	Rene Sola	1.8	1.6

WST company power—a foreign-owned, high-tech PV enterprise with research and development, production, and sales—is mainly engaged in solar cells; components of crystalline silicon; solar cells with thin film; photovoltaic power generation systems; and the research and development, manufacturing, and sales of BIPV products, and also its business is all over the world. WST company was founded in 2001, listed on the New York Stock Exchange in 2005, and was bankrupt and restructured in 2013. The WST company went from nothing in a relatively short period of time and exceeded Western and Japanese manufacturers to be one of the world’s overlords; then, as good times do not last long, it fell into the morass of stagnant growth until bankruptcy reorganization and, finally, was newborn after the fire. What are the factors making it the top spot in the PV industry, and what factors forced it to suffer from boom to bust and to bankruptcy? We’ve made a review from four aspects of technology research and development, capacity scale, project cooperation, and resource integration.

**FIGURE 4:**
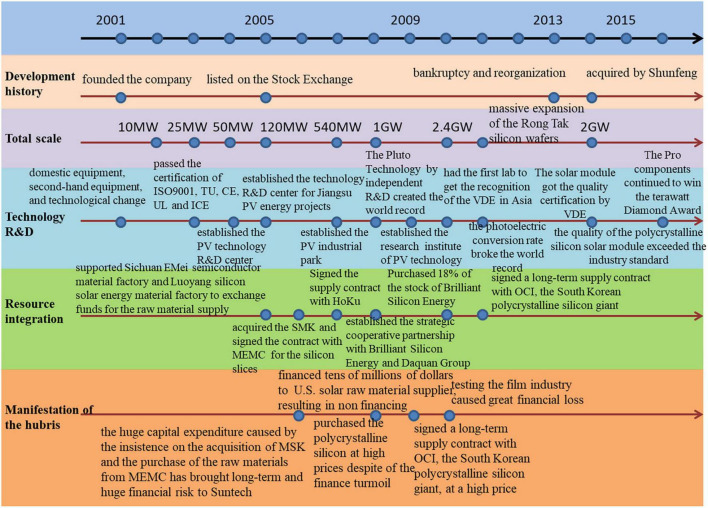
The development of WST company from year 2001 to 2016.

We summarize the developmental stages of the case company in Figure 4.

## Findings and Discussion

The dynamic capability–building process of an energy enterprise is reported in this article. This part regroups over the materials based on the case material by utilizing table form, according to the time sequence as well as taking the development of WST company as the axes. The key concept is classified and analyzed separately so that we can see the start-up, development, decline, and redevelopment of the dynamic capability–building process. The ambidexterity emphasis of each stage and the impact made by the managers’ hubris over the dynamic capability and stage target are also investigated. We summarize these findings in Table 2.

**TABLE 2:** Evidences of the major constructs emerged.

	First stage (before 2003)	Second stage (2003)	Third stage (2006)	Fourth stage (2008)	Fifth stage (2009)	Sixth stage (2010)	Seventh stage (2011)	Eighth stage 2012–2013	Ninth stage (2014–2016)
Exploitation/Routine		(1) Continuous expansion of the production lines (2) applied for the international certification for the techniques (3) continued to exploit the markets home and abroad	(1) got the government support in policies, funding, and projects (2) established the business alliance with the manufacturers of raw materials (Sichuan Emei, Henan Luoyang, Jiangsu Jintan, and Guangzhou aluminum paste factory) (3) conducted the technical cooperation with the forty-eighth Research Institute of the Ministry of information industry (4) established a joint venture with Jiangsu Jintan, to produce silicon bars (5) exchanged the capital for the silicon raw materials from MEMC	(1) formed the strategic cooperative partnership with Brilliant Silicon Energy and Daquan Group and also signed the contract for the polycrystalline silicon supply (2) WST company and Yingli signed a cooperation agreement (3) on the basis of 3 overseas R&D centers in Germany, Japan and Australia, established WST company photovoltaic technology research institute (4) constant update on the techniques (5) cooperated with the United States supplier of THE solar energy raw materials and made financing	(1) participated in several government projects (2) signed cooperation agreements with other companies	(1) the techniques continued to lead, and its laboratory for the photovoltaic products inspection won VDE recognition (2) made production expansion, and set up the production base in Minhang District, Shanghai (3) had three major overseas R&D bases in Japan, Australia, and Germany (4) introduced the CSF (Fund) investment (5) built a manufacturing plant with 30 megawatt photovoltaic module in Phoenix, Arizona	(1) continued overseas market expansion, WST company’s largest photovoltaic power plant project components in Southeast Asia officially launched (2) continued to expand the production (3) technology continued to lead, the polycrystalline silicon and the monocrystalline silicon solar cells broke the world record of the photoelectric conversion efficiency of the crystalline silicon solar cells at that time		(1) the manufacture and sale of the solar panels (2) the technology continued to be renewed, and the polycrystalline silicon solar modules were considered to be above the industry standards in the technical assessments organized by OST Energy, a well-known research institution in the United Kingdom. WST company’s solar modules had been certified by the VDE quality test. A new generation of the high-efficient components for the test authentication had been developed (3) continued to work with overseas customers; WST company worked with Adani power, one of India’s largest privately- owned power producers
Exploration/*ad hoc*	(1) the first production line successfully debugged (2) opened up the international market (3) paid attention to the technology research and development (4) participated in the international exhibition of the new energy products	(1) raised funds with the help of the government and obtained the policy support (2) constructed the qualified personnel tams and scientific research groups (3) established domestic R&D centers (4) supported Sichuan EMei semiconductor material factory and Luoyang silicon solar energy material factory to exchange funds for the raw material supply	(1) acquisition of MSK company	(1) faced with financial turmoil, cut down the production line and laid off (2) WST company pledged to invest 258 million Euros to GSF (fund) and obtained 86% of the stake					(1) carried out the power generation business and vertically integrated services related to the photovoltaic power station construction (2) signed the cooperation agreements with Munich re -insurance company and Ping An Property Insurance Company of China (3) led the drafting of two SEMI standards, while participating in the setting of multinomial standards
Managerial Hubris	none	none	the huge capital expenditure caused by the insistence on the acquisition of MSK and the purchase of the raw materials from MEMC had brought long-term and huge financial risk to WST company	(1) bought the polycrystalline silicon at a high price despite of the financial turmoil (2) financed tens of millions of dollars to United States solar raw material supplier, resulting in nonfinancing	(1) signed a long-term supply contract with OCI, the South Korean polycrystalline silicon giant, at a high price	ShiZhengrong hoped to test the film industry through the R&D of the thin film cell and the establishment of the base, but the conversion efficiency of the thin film cell was far lower than that of the United States counterparts, then WST company decided to stop the project in the first half year of 2010, resulting in a loss of $50∼$55million	(1) WST company signed another long-term supply contract with OCI, the South Korean polycrystalline giant		none
Ambidexterity or not	More Exploration	Ambidexterity	More Exploitation	More Exploitation	Exploitation	Exploitation	Exploitation		Ambidexterity

At the initial period of the pioneering work, before the year 2003, the first production line was successfully debugged by WST company, which shortened the difference between the international photovoltaic industry and that of our country simultaneously for nearly 5 years and reached the advanced level of the world’s counterparts. As there was no domestic marketing growing, Doctor Shizhengrong located the sales targets to the overseas market, and he was removing from different countries, such as Germany, Japan, Holland, and South Africa, for the whole 4 months and expanding the international market. He highly stressed technical research development and obtained a great hit in participating in the International New Energy Products Fair. WST company at this moment was at the probing and starting period.

During the period of 2003–2005, WST company made a continuous expansion over the solar energy production line depending on the previous route. A lot of energy was spent on applying for international technical certification after it was put into operation, which obtained almost all the international pass certifications in the solar energy industry, such as ISO9001, TU, CE, UL, IEC, etc., and cleaned up the barriers for opening the international market. Additionally, WST company carried out new explorations and trials, established a domestic research center, organized a professional scientific research group, and valued the construction of a talent group. Furthermore, it also raised funds domestically by using the support of the municipal local government and sponsoring the semiconductor material factory of Emei Sichuan and silicon solar material plant of Luoyang so as to exchange the raw material supply with the technology.

According to the definition of O’Reilly, exploitation refers to the activities made by the enterprise by utilizing existing knowledge and skills to improve the products and services within current operating activity domains. Exploration means the activities made by the enterprise by getting rid of the current operating domains and utilizing new knowledge and skills to find new marketing opportunities as well as expand the new marketing space (O’Reilly and Tushman, 2008). It can be seen that WST company not only updated the expansion and technology according to the route dependence during 2003–2005, meanwhile, it also explored new domains. Such capacity, which is committed to exploitation and exploration, is called the ambidexterity capacity of the organization (Duncan, 1976; Tushman and O’Reilly, 1996).

In 2003, WST company obtained sales revenue of 1.5 billion RMB with a profit of 1.2 million RMB, and the production and operation were on track. At the beginning of 2004, after evaluating the time and situation carefully, Doctor Shi firmly proposed to the board of directors again to expand 25 megawatts. His proposition obtained approval again, and a third production line was put into operation smoothly in August 2004, which realized an annual production capacity of 50 megawatts, ranking first in the country and 10th in the world. In August of 2004, his proposition of expanding a 50-megawatt battery line was approved again by the board of directors of the company. Therefore, 107 acres of land was purchased by the company in the new district of local government, and two new lines were put into operation in July 2005.

The smooth operation and feedback with high targets of the company during these continuous several years not only enhanced the company to be on track gradually, but it also made the founder feel more confident in the company’s development. The expansion proposals each time were put forward by him personally, and they all obtained approval. A series of successes drove the burning of the fire full of hubris and pride from the heart of the founder. As the scholars say, Hayward and Hambrick (1997) propose that the recent success of the company was the main reason that led to the hubris of the managers.

From 2006 to 2007, the proportion of the development aspect was enlarged, and it continuously obtained support from government policy, funds, and projects and established enterprise allies with the raw material manufacturing factory (Sichuan Ermei, Henan Luoyang, Jintan of Jiangsu Province, aluminum pulp mill of Guangzhou). Furthermore, it had technological cooperation with the No. 48 institution of the national information industry department and set up a joint venture to produce silicon rods with Jintan of Jiangsu province. In the aspect of exploration, it acquired MSK. But, from this moment, the founder started to expose his inclination of managerial hubris. On August 2, 2006, the WST company solar energy electricity limited company successfully acquired MSK, the third biggest professional solar energy panel production enterprise in Japan, which concerned about 300 million U.S. dollars. A week before acquiring MSK, the founder decided that WST company was going to sign a silicon wafer supply contract with MEMC, one of the biggest global silicon wafer suppliers, listed on the main board of the New York Stock Exchange. According to later observation, the huge capital spending of acquiring MSK company and purchasing the silicon raw material of MEMC bought huge, long-term financial risks to WST company. The CEO with hubris generally blocks the emergence of feasible ideas (Shipman and Mumford, 2011) and ignores them to make any adjustment to stimulate the organization so as to respond to critical feedback.

The founder did research on crystalline silicon thin film solar batteries by learning from Professor Martin Green, the international solar battery authority and the Nobel Prize winner for the environment in 2002. He was the first one who overcame a huge difficult issue on how to grow a silicon film on glass and obtained more than 10 invention patents for solar energy battery technology. From the founder’s resume, it can be seen that he was a person with high qualifications and strong professional competence, which complies with the concept of the research findings of Bhandari and Deaves (2006) that males with a deeper educational background and higher qualifications are more likely to develop hubris and arrogance than those counterparts with less educational experiences.

Except for some personal factors, the relevant documents about hubris and arrogance also list some environmental factors about the hubris source of the managers: (1) recent success of the company, (2) compliments of the recent media, (3) arrogance of the CEO, and (4) the sense of power of the CEO in a long-term authoritative position.

Recent media evaluation strengthened the position of the CEO through the compliments on the targets and enlarging the effects and control of the CEO. Meanwhile, the phenomenon of the celebrity effects were also formed.

In April of 2006, Li Yuanchao, the Party Committee Secretary of Jiangsu Province of that time, inspected the solar energy of the city, and he gave instructions to Yang Weize, the accompanying Party Secretary of the city on the scene, requiring that the city must play a leading role in introducing overseas talents in the whole province, enlarging the “effect of WST company” as well as batch copying the “pattern of the founder.” The implementation plan: The municipal government gives 1 million RMB as start-up capital to the entrepreneur, a working site no less than 100 m^2^, and a living flat no less than 100 m^2^. The two latter items can be free of rent within 3 years. It should also provide a venture capital fund of no less than 3 million RMB and commercial loan guarantee of no less than 3 million RMB during the industrialization process. The success of WST company within a short-term period popularized the founder. The governmental leading and policy tendencies enlarged WST company’s effects and celebrity effects. The media compliments became one of the most significant sources for creating the later hubris of the founder. Besides this, the founder, as a founder, took such an authoritative position as CEO in WST company all time, and his very important position in the company also caused his cognitive errors of the managerial hubris.

The individual influenced by the hubris and arrogance generally shows a kind of self-dignity of a high degree and unrealistic optimism, and they think too much about their self-knowledge and capacity. Meanwhile, they believe that their performance is better and more efficient than other people’s (Picone et al., 2014).

The founder himself majored in thin film batteries when he was studying for a doctoral degree in Australia, and familiar people revealed that he also hoped to test this industry through establishing a research, development, and manufacturing base in Shanghai in 2007. However, actually, the thin film batteries base stopped developing with very small output. Besides this, the exchange efficiency of the WST company thin film battery was only 7%, which was much lower than the American counterpart levels by 11–12%. Therefore, WST company decided to stop this project in the first half year of 2010, which caused a loss of about 50 million or 55 million U.S. dollars. Furthermore, one of the identities of the founder was as the “international consultant” of the NYSE with only 30 people having this position globally.

The qualifications and achievements obtained by the founder led to his great confidence over his own professional competence and knowledge, which made him take a great optimistic attitude toward his major: thin film batteries and testing this industry. Such self-arrogance caused the great loss of this project. A CEO with overconfidence tends to overestimate the ability to solve the problems of other people.

In 2008, WST company still emphasized development and utilization and cooperated with many enterprises, which formed the strategic cooperative partnership with Brilliant Silicon Energy and Daqo Group by signing a supply contract with Polysilicon and a cooperative agreement with Yingli Green Energy. It also paid close attention to the aspects of research and development. WST company Photovoltaic Technology Research Institute was set up on the basis of three overseas research and development centers in Germany, Japan, and Australia. In the aspect of exploration, the production line cut production and laid off employees in face of the financial storm in 2008. WST company promised to invest 258 million euros on the GSF fund and obtained equity interests of 86%.

Since the financial crisis started to spread in the middle of September 2008, although the orders of WST company did not seem to decrease, the payment had already become a big problem. Some clients were not able to issue letters of credit from the bank, or the letter of credit that used to be issued for 2 days in the past could not even be issued for 2 weeks or even 2 months, which was already a symptom of the extreme shrinking of needs. It would have been proper behavior to reduce the raw material purchase, but the founder took a completely different action. To ensure the production for four quarters, WST company purchased part of Polysilicon with a price range of 350–400 U.S. dollars per kilogram (which is slightly higher than that in July or August) in September 2008; he underestimated the uncertainty of the market and ignored the risks of the financial storm. However, in October, the price of the silicon material was plummeting with a more than 50% decline, which foreshadowed the huge decline of gross profit for the four quarters of WST company. The behavioral decision theory thinks that the hubris or overconfidence, as a form of awareness prejudice, promoted the decision maker to overestimate his ability to solve problems (Camerer and Lovallo, 1999) and underestimate the resource needs of the risky projects (Shane and Stuart, 2002) as well as underestimate the uncertainty confronting the company.

At the beginning of 2011, WST company signed another long-term supply contract with OCI, the Korean polysilicon giant, which regulated that WST company would purchase with the price more than 30 U.S. dollars per kilogram. It can be found by contacting the previous contract signatures that WST company used to pay a high price. Hayward and Hambrick (1997) reveal in the previous research that the hubris drives a CEO to pay higher extra fees during mergers and acquisitions.

From 2012 to 2013, WST company confronted bankruptcy. On August 15, 2012, the founder was relieved as CEO, serving as the executive director and chief strategy officer of the company. The former CFO, Jinwei, served as CEO of the company. On March 5, 2013, after being relieved of the CEO position, the founder was also relieved of the position of the director of WST company by the board of directors and formally lost control power over WST company. On November 4, 2013, Jiangsu wind photoelectric international limited company listed in Hong Kong successfully acquired WST company solar energy electricity limited company with the price of 300 million RMB. Lowe and Ziedonis (2006) think that overconfident entrepreneurs are more likely to cause the bad targets of the company. Hayward et al. (2006) indicates that overconfident entrepreneurs are more likely to lead to the failure of their enterprise. The overconfidence likely produces overambitious strategies and exaggerates the need for decisiveness, impulse, and power as well as the risks of spontaneity. All these behaviors result in catastrophic consequences (Picone et al., 2014).

In January 2014, the bankruptcy restructuring case of WST company was concluded; the alternation work of the legal representative was also finished on February 2014. Wang Xiangfu, CEO of Shunfeng Photovoltaic replaced the founder to serve as the legal representative of WST company. From 2014 to the present, WST company has recovered to produce due to the alternation of the managing layer and the cooperative agreement signed by the Munich Reinsurance Company and China Pin property insurance. It continues to manufacture and sell solar energy battery panels and updates the technology continuously. Polysilicon solar panels was authenticated that the quality was over the industry standard by the technical evaluation of a well-known British research institution, OST Energy organization. The solar panels of WST company also obtained VDE quality test authentication; it also carried out the power generation and vertical integration business related to photovoltaic station construction and led to drafting two copies of the SEMI standard; meanwhile, it also participated in the stipulation of various standards. The decision right during this time was no longer controlled by a single person as it was before.

Let us look at the operating income situation of WST company from 2002 to 2011. Generally speaking, the entire operating income tends to be rising except for 2009, but the growth rate status is presented as rising rapidly and then falling down dramatically and slowly. It rose again a little bit in 2010 and fell down again in 2011.

From 2002 to 2005, there was no phenomenon of manager hubris appearing in the company, especially during 2003–2005. WST company was not only able to utilize existing knowledge and skills to improve the product and service within the current operation activity domains, but also utilized the new knowledge and skills to discover new market opportunities and expand new marketing spaces by getting rid of the current operation domains. Although the operating income during this period was not too much, it was growing very rapidly.

**FIGURE 5:**
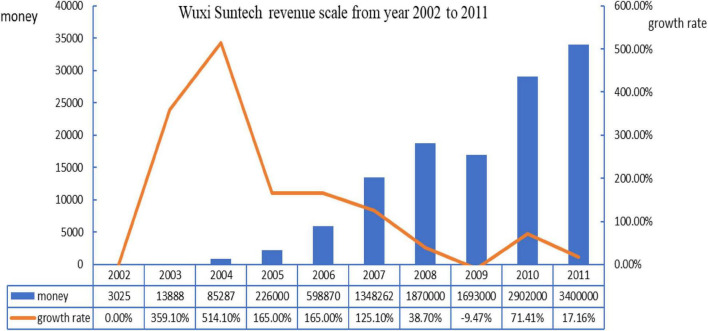
WST company revenue scale from year 2002 to 2011.

From 2011 to 2016, the phenomenon of the manager’s hubris came into being in WST company. The founder made a series of wrong decisions due to his hubris and arrogance by overestimating his own abilities so that it brought a huge financial loss to WST company. During this period, WST company began to emphasize the development and utilization from previous ambidexterity in the business. Although the gross revenue during this period was still inclined to be rising, the growth rate was obviously lower than that before 2006 with a consecutive 3-year decline. The new lowest record in history even appeared in 2009. It was rising back a little bit in 2010 and was falling down again in 2011. We can see the WST company revenue scale from year 2002 to 2011 in Figure 5.

## Discussion

We can see from the previous analysis that WST company was in the full exploration stage before 2003 because the company was just founded at this time and needed to open up the market and business. From 2003 to 2005, WST company owned ambidexterity, including not only the exploitation stage, but also the exploration stage. There is no phenomenon of the manager’s hubris at this stage, and the company landed on the United States Nasdaq and was in a period of rapid growth. From 2006 to 2008, although having both the exploitation and exploration, the company put more emphasis on the former, and there was the phenomenon of the manager’s arrogance at this stage. From 2009 to 2011, WST company was in the full exploitation stage, accompanied by the manager’s arrogance in the first 2 years. The growth rate in 2009 is a new lowest record in history. In 2011, WST company has no phenomenon of the manager’s arrogance, but at this stage, WST company is already in deep crisis. The company’s profits were basically in steady growth until the year before the bankruptcy (2011). From 2012 to 2013, WST company conduct bankruptcy reorganization. From 2012 to 2016, the management layer made frequent changes. From 2014 to 2016, the recombinant WST company restored ambidexterity to continue to gain both and return to the right track.

From here, we can see that, when the manager’s hubris appears, the enterprise is not able to take into account both exploitation and exploration abilities, which leads to the weakness of the dynamic capability; on the contrary, when the manager’s hubris gets weak, namely, rights are dispersed, the enterprise can take into account both exploitation and exploration, and dynamic capability is enhanced.

For the company’s performance, when the manager’s hubris appears, namely, from 2006 to 2010, the founder’s hubris led to the wrong decisions, resulting in the stage performance loss to different degrees. For example, in 2006, the huge capital expenditure caused by the insistence on the acquisition of MSK and the purchase of the raw materials from MEMC brought long-term and huge financial risk to WST company. In 2008, WST company raised tens of millions of dollars for the United States solar energy raw material supplier, making it impossible to draw back. In 2009, WST company signed a long-term supply contract with South Korea’s polycrystalline silicon giant OCI at a high price. In 2010, the founder himself hoped to test the film industry through R&D of the thin film cell and the establishment of the base, but the conversion efficiency of the thin film cell is far lower than that of the United States counterparts; then, WST company decides to stop the project in the first half year of 2010, resulting in a loss of $50∼$55 million.

When the decision making becomes arbitrary, the manager gets high self-esteem, leading to a low degree of ambidexterity, and then, the dynamic capability cannot be constructed, and therefore, environmental changes cannot be responded to, resulting in a low performance at this stage. When the rights get dispersed, the manager gets low self-esteem; thus they can build dynamic capability, and corporate performance rises.

## Conclusion and Implications

Previous studies generally only discuss how dynamic capability is constructed, and few scholars study how dynamic capability is constructed, falls, and rises again. This paper extends the cycle perspective of dynamic capability research, which provides a more detailed understanding of the dynamic development of the dynamic capability itself.

Second, this paper puts managerial hubris into the entrepreneurial environment to consider, discussing its influence on the development of dynamic capability and its impact on the company’s performance, which is a new supplement to the previous study, and makes a contribution to the theoretical research on managerial hubris and also has good reference significance for the company’s development at the same time.

For theoretical implications, this study points out clearly that research needs to explore for microfoundation of the firm-level dynamic capability evolution. Also, such exploration does not necessarily need to be conducted from a positive lens. Although preliminary, this study shows that dynamic capability may evolve in a negative way, especially in the entrepreneurial context, that is affected by microlevel factors. Future studies are encouraged to link important individual and/or group factors, such as personality, value, political skills, etc., of entrepreneurs or entrepreneurial teams to the positive or negative evolution of dynamic capability.

For practical implications, we suggest that it is always important to make clear assessment of the influences of an entrepreneur/entrepreneurial team’s microlevel traits on the development of a new venture’s competitive capabilities. Such assessment may not be done by the traditional HR department due to the special characteristics of an entrepreneurial venture. Potentially, the members of the entrepreneurial team themselves may be the best to monitor such traits of one another to reflect on dynamic capability development. In the case of a single founder, it is a challenge for there might be a lack of an appropriate mechanism for founder traits and behaviors, especially in some cultural context such as the one studied here. With this in mind, future studies are also suggested to explore appropriate governance mechanisms for guiding the individual–new venture capability relationships.

## Data Availability Statement

The original contributions presented in the study are included in the article/supplementary material, further inquiries can be directed to the corresponding author/s.

## Ethics Statement

The studies involving human participants were reviewed and approved by Tianjin University, China. The patients/participants provided their written informed consent to participate in this study.

## Author Contributions

S-CF conceived and designed the research and provided guidance throughout the entire research process. YG and CC wrote the original manuscript. P-WH processed and analyzed the data. F-ST reviewed and edited the manuscript and was responsible for all R&R works. All authors contributed to the article and approved the submitted version.

## Conflict of Interest

The authors declare that the research was conducted in the absence of any commercial or financial relationships that could be construed as a potential conflict of interest.

## Publisher’s Note

All claims expressed in this article are solely those of the authors and do not necessarily represent those of their affiliated organizations, or those of the publisher, the editors and the reviewers. Any product that may be evaluated in this article, or claim that may be made by its manufacturer, is not guaranteed or endorsed by the publisher.
